# Overall View of Chemical and Biochemical Weapons

**DOI:** 10.3390/toxins6061761

**Published:** 2014-06-04

**Authors:** Vladimír Pitschmann

**Affiliations:** 1 Faculty of Biomedical Engineering, Czech Technical University in Prague, nám. Sítná 3105, Kladno 27201, Czech Republic; 2 Oritest spol. s r.o., Staropramenná 17, Prague 15000, Czech Republic; E-Mail: pitschmann@oritest.cz; Tel.: +420-257-311-639

**Keywords:** chemical weapons, biochemical weapons, Chemical Weapons Convention, methods of chemical weapons development

## Abstract

This article describes a brief history of chemical warfare, which culminated in the signing of the Chemical Weapons Convention. It describes the current level of chemical weapons and the risk of using them. Furthermore, some traditional technology for the development of chemical weapons, such as increasing toxicity, methods of overcoming chemical protection, research on natural toxins or the introduction of binary technology, has been described. In accordance with many parameters, chemical weapons based on traditional technologies have achieved the limit of their development. There is, however, a big potential of their further development based on the most recent knowledge of modern scientific and technical disciplines, particularly at the boundary of chemistry and biology. The risk is even higher due to the fact that already, today, there is a general acceptance of the development of non-lethal chemical weapons at a technologically higher level. In the future, the chemical arsenal will be based on the accumulation of important information from the fields of chemical, biological and toxin weapons. Data banks obtained in this way will be hardly accessible and the risk of their materialization will persist.

## 1. Introduction

The Convention on the Prohibition of the Development, Production, Stockpiling and Use of Chemical Weapons and their Destruction (Chemical Weapons Convention, CWC) was submitted for signing in Paris on 13 January 1993, after complicated negotiations. The Convention came into effect on 29 April 1997, and up to the present time (31 March 2014), 190 countries have participated in the Convention. It has been signed, but it has not yet been ratified by two countries (Israel, Myanmar); and, four countries have not yet signed the Convention (Angola, Egypt, South Sudan, the Korean People’s Democratic Republic). In accordance with Article I, each contractual country undertakes not to develop, produce, otherwise acquire, accumulate, keep and transfer chemical weapons in any situation. The contractual country is obliged not to use chemical weapons, not to perform any preparation for their military use and to destruct its own chemical weapons and the facilities for their production that are under its jurisdiction and control. It also undertakes the destruction of all the chemical weapons left in the territory of the other contractual country.

A total of about 71,000 tons of chemical weapons have been declared (chemical toxic substances and precursors). The largest stocks were declared by Russia (40,000 tons) and the United States (30,000 tons), the remaining proportion by Albania, India, Iraq, South Korea, Libya and, recently, also, Syria. In accordance with the original intention, the chemical weapons were supposed to be destroyed within ten years after the CWC came into force, *i.e.*, by the end of April, 2007. This has not been the case, and thus, the Organization for the Prohibition of Chemical Weapons (OPCW) in Haag adopted an extension of the term for the destruction of all of the declared stocks by a further five years. By 2012, more than 51,000 tons of chemical weapons had been destroyed, which is about 72% of the stocks declared. Thus, the destruction of chemical weapons is still under way. All of the stocks of Albania, India and South Korea have been destroyed. As a yet unresolved question, the ownership of chemical weapons is suspected for countries that have not yet become participants in the CWC. There is also an open question about the destruction of old and abandoned chemical weapons and about chemical weapons sunken in the sea or deposited under the ground.

Thus, it seems that the era of chemical weapons as one of the traditional technologies for weapons of mass destruction has been terminated. Some authors consider chemical weapons to be directly consigned to the scrap heap of history [1]. On the other hand, over the last twenty years, a number of serious arguments have been presented that offer a less optimistic view. The aim of the present work is to attempt to answer several basic questions: Is the existence of chemical weapons associated with a particular historical era? Are the reasons that resulted in adopting the CWC still persisting? What is the currently existing technical level of chemical weapons? Is there a scientific-technical potential for their further development?

## 2. Brief History of Chemical Weapons

### 2.1. Prehistory and Pre-Industrial Era

The use of toxic substances as weapons has a long history of several thousands of years. It is associated with traditional methods of hunting, including the use of poisoned weapons (arrows, spears), the poisoning of water (watering holes, fishing) or the fumigation of animals with toxic combustion products. All of these hunting forms using toxic substances, which also appeared in combat and which were later particularly developed for ancient wars, have persisted until present time. The Middle Ages saw the enrichment o military technology with the discovery of gunpowder (including its prototype, “Greek fire”), which made possible not only the development of new weapon systems, but also new methods for the transfer of toxic substances to warfare conditions [2]. This stepwise development continued from the first Chinese primitive chemical grenades, still launched by mechanical artillery, to chemical ammunition of an advanced type, which occurred as soon as at the beginning of the industrial era. For example, at the time of the Crimean War, in addition to a proposal for taking Sevastopol with the help of toxic sulfur dioxide obtained by the combustion of sulfur, there was also a design for artillery shells filled with cacodyl cyanide. In the American Civil War, there were also proposals of using artillery shells filled with chlorine, hydrogen cyanide (a binary system consisting of potassium cyanide and hydrochloric acid), compounds of arsenic or poisonous plant material (for example, *Capsicum*, *Piper*, *Veratrum*) [3].

### 2.2. Onset of Chemical Weapons in World War I

A decisive break in the history of chemical weapons was encountered in World War I, particularly after the German chlorine attack in Ypres on 22 April 1915. This fact can be explained by a combination of several basic conditions, such as the enormous extent of the war conflict, the high level of science and technology, the necessary capacities and the as yet experienced development of the chemical industry. The immediate stimulus for the mass development and use of chemical weapons was the attempt of field armies to solve the acute lack of conventional ammunition and to react to the reality of static (trench) warfare, which was developed on all of the main fronts. Chemical weapons were expected to break the defense of the enemy, to establish conditions for a conversion to mobile warfare and, thus, to contribute to the final victory. This strategy was definitely not fulfilled, but chemical weapons demonstrated their high efficacy and became an important operational-tactical factor. They were essentially used in any large combat, and their proportion of the total volume of the weapons used was dynamically increased. The main big powers (Germany, France, Britannia, Russia and the United States) produced more than 200,000 tons of chemical warfare agents (CWA), and out of this, the largest amounts were used by all the methods known at that time (gas cylinders, tube artillery, mortars, “gas projectors”, hand grenades and rifle cartridges). So-called rapidly acting lethal substances had a precedence (chlorine, phosgene, diphosgene, chloropicrin, hydrogen cyanide); and, at the last stage, also vesicants (mustard gas). Most land operations were accompanied by the use of harassing agents (tear gases, irritating aerosols). Chemical weapons eliminated about one million soldiers from action, and about 10% of them died. This was also of a considerable economic and logistic importance. In addition, chemical weapons demonstrated high psychological effects, deepened the privations of military troops and enhanced the fighting effects of conventional weapons [4,5,6].

### 2.3. Continuing Development of Chemical Weapons

After World War I, public opinion definitely refused the use of chemical weapons, seeing them as inhumane, but chemical warfare had already become an integral part of military doctrines. So-called first generation chemical weapons were employed by both parties in the civil war in Russia, by the Spanish and French armies in Morocco and, later, also, by Italian troops in Abyssinia and by the Japanese army in Manchuria. The air force, equipped with chemical bombs and equipment for spraying, able to penetrate deep into the enemy background and to endanger large cities and industrial complexes, became a new element, disrupting the strategic equilibrium. Chemical arsenals were extended by further CWA. The most important group of vesicants still (mustard, lewisite) was supplemented by propylene mustard, O-mustard, sesquimustard, numerous variants of nitrogen mustard and different tactical mixtures. The development of chemical weapons in the interwar period achieved its maximum in the late 1930s. Chemists of the German IG Farben synthesized a series of new organophosphates within the framework of the research and development of pesticides, which are effective inhibitors of acetylcholine esterase (AChE) [7]. Nerve agents, named tabun and sarin, became the basis of chemical weapons of the so-called second generation.

### 2.4. Chemical Weapons in World War II

In the course of World War II, big powers exerted technical, as well as organizational readiness to use chemical weapons on a mass scale [8]. This is supported by the fact that they stored above 400,000 tons of CWA; out of this, 2/3 were vesicants (mustard, lewisite). However, until 1944, Germany also produced considerable stocks of chemical weapons of the second generation, *i.e.*, more than 12,000 tons of tabun and a smaller amount of sarin. It is thus of interest that the expected total chemical warfare did not occur; only the Japanese army employed chemical weapons to a limited extent (even if with serious consequences) in China. There are several possible explanations. One reason could be the personal negative experiences of Adolf Hitler, who was, at the time of World War I, seriously affected by mustard gas. At the first stage of the successful blitzkrieg, Germany did not need chemical weapons. Later, when they lost the strategic initiative and started seriously considering the use of chemical weapons, the fear of retaliation attacks was prevalent. The Allies repeatedly discouraged Germany (but also Japan) with their chemical arsenal, and they probably would not have hesitated to use it if necessary. If chemical warfare began in Europe, the Allies would take advantage of the prevalence in the absolute volume of their chemical weapons. On Germany’s part, there was the advantage of stocks of chemical weapons of the second generation, their existence being most likely unknown to the intelligence services of the Allies. A special chapter of World War II history is the use of carbon monoxide and hydrogen cyanide (Zyklon B) for the extermination of Jewish people and other nations in concentration camps or possibly chemical-toxicological experiments on humans.

### 2.5. Chemical Weapons in Nuclear Age

After World War II, there was a high precedence of the development of strategic arsenals based on nuclear technology, but there were also qualitative changes in the development of chemical weapons [9]. A characteristic feature of the first period of the Cold War was the introduction and mass manufacture of G nerve agents (sarin, soman) and the supplementation of chemical weapons of the second generation by even more effective V agents (VX, R-33). More modern tools for chemical attack also occurred, including tube, as well as rocket artillery, air force and ballistic missiles. At the dawn of the 1950s and 1960s, new concepts of conducting “non-lethal” (psychochemical) war was elaborated with the use of psychoactive substances, particularly hallucinogenic glycolates (BZ). In the arsenal of armies, as well as of police and security services, new highly effective irritants arose, which were also employed in military conflicts. The best known example is the mass use of the CS agent in the Vietnam War. American forces concurrently used herbicides on a large scale (particularly based on 2,4-D and 2,4,5-T), the primary purpose of this being the vertical and horizontal control of the vegetation on the battlefield. The mass application of herbicides exerted negative effects on the environment, agriculture and health conditions of the local population [10,11]. In the peak period of the Cold War, the United States developed chemical weapons of the third generation, the principle of which was binary ammunition based on nerve agents (sarin, VX, IVA (intermediate volatility agents)). The Soviet Union reacted with an extensive program (code name FOLIANT, NOVICHOK) for the development of new, fourth generation chemical weapons, and the result was a technology for binary ammunition with nerve agents exerting enhanced toxicity. In the period of the Cold War, chemical weapons were also distributed into countries of the so-called Third World, which considered this technology to be a cheaper alternative to nuclear weapons. The largest advance in this direction was achieved by Iraq, where a chemical arsenal, including more than 3,800 tons of CWA (mustard, tabun, sarin), was accumulated with the help of foreign companies. A major proportion of these stocks were used in the war against Iran and against the local population of Kurds [12]. For an outline of the most important classic CWA and their classification into particular chemical weapons generations, see Table 1 and Table 2.

**Table 1:** Overview of the most classic chemical warfare agents (CWA).

Agent	Code name	Chemical name	Molecular weight
Sulfur mustard	H, HD	Bis(2-chloroethyl)sulfide	159.1
Nitrogen mustard	HN-3	Tris(2-chloroethyl)amine	204.5
Lewisite	L	Dichlor(2-chlorovinyl)arsane	207.3
Tabun	GA	Ethyl-(dimethylphosphoramido)cyanidate	162.1
Sarin	GB	Isopropyl-methylphosphonofluoridate	140.1
Soman	GD	(3,3-dimethylbutan-2-yl)-methylphosphonofluoridate	182.2
Cyclosarin	GF	Cyclohexyl-methylphosphonofluoridate	180.2
VX agent	VX	S-[(2-diisopropylamino)ethyl]-O-ethyl-methylphosphonothiolate	267.4
R-33 agent	R-33	S-[(2-diethylamino)ethyl]-O-isobutyl-methylphosphonothiolate	267.4
Chloroacetophenone	CN	ω-Chloroacetophenone	154.6
CS agent	CS	2-Chlorobenzylidene malononitrile	188.6
CR agent	CR	Dibenz[*b,f*]-1,4-oxazepine	195.2
BZ agent	BZ	3-Quinuclidinyl benzilate	337.4

**Table 2:** Generations of classical lethal chemical weapons.

Generation	Chemical Weapons (CWA)	Biological Effects	Examples	Note
1	Choking agents	They attack lung tissue, primarily causing pulmonary edema	Phosgene, diphosgene, chloropicrin	WW I
	Blood agents	Affect the bodily functions by inactivating the cytochrome oxidase system	Hydrogen cyanide, cyanogen chloride	WW I
	Blister agents	They cause inflammation, blisters, and total destruction of tissue	HD, L / HN-1,2,3	WWI/1930s
2	Nerve agents G	Nerve agents disrupt the functions of the nervous system by interfering with the enzyme, AChE	GA, GB, GD	WW II
	Nerve agents V	As nerve agents G	VX, R-33	1950s–1960s
3	Binary	As nerve agents	GB-2, VX-2, IVA-2	1970s–1980s
4	Binary NOVICHOK	As nerve agents	A-230, A-232, A-234	1980s–1990s

## 3. Reasons for Adopting the CWC and Contemporary Risks of the Use of Chemical Weapons

### 3.1. Human Reasons for Adopting CWC

One of the targets of any disarmament action is establishing certain general humanity principles. This also concerns the prohibition of chemical weapons. As early as 1868, a special international military commission in St. Petersburg elaborated a declaration stating that the use of weapons, which bring useless enhancement of the suffering of people or unavoidable death, is against humanity rules. In 1874, a conference was held in Brussels declaring that the war right does not provide unlimited freedom in choosing tools for the destruction of the enemy. Based on this declaration, the conference refuses the use of poisons and poisoned weapons, projectiles and agents, which could cause useless suffering. Given these humanity principles, the Convention Respecting the Laws and Customs of War on Land was elaborated in Haag from 1899 to 1907, prohibiting the use of poisons or poisoned weapons and also weapons, projectiles or agents that are able to cause useless suffering. There was an analogous prohibition of the use of projectiles having as their only target the propagation of choking or harmful agents. In the course of World War I, no declarations or rules were able to prevent the mass use of chemical weapons. Due to this, based on a resolution of the UN General Assembly, a disarmament conference was arranged in Geneva, which elaborated an agreement of the prohibition of using choking, poisonous or similar gases, liquids, agents or similar means in war and also the prohibition of the use of bacteriological methods in conducting war. This Geneva Protocol, submitted for signing in 1925, has still been an important part of international law, and the CWC also refers to it in its preamble.

### 3.2. Military-Technological Reasons for Adopting CWC

An important reason for most disarmament initiatives was also the attempts of big powers to cope with the progress of the enemy or to eliminate the lack of success in the development of the technology of a certain type of weapon. For example, in the 1960s, the big powers concluded that biological weapons (particularly pathogenic agents) are not practicable for military purposes, since their effects are quite beyond any control. The result of this was the Convention on the Prohibition of the Development, Production and Stockpiling of Bacteriological/Biological and Toxin Weapons and on Their Destruction (Biological and Toxin Weapons Convention, BTWC), which was signed in Geneva in 1972 and came into force three years later. As a drawback of the Convention, there is, however, the lack of a control mechanism. One of reasons for this is that the principle of biological weapons and their military use have not yet been unambiguously delimited.

There were no principal doubts about the perspective of chemical weapons in contrast to biological (especially bacteriological) weapons. In spite of this, at the dawn of the 1970s and 1980s, a number of problems were encountered. That time, the United States owned a rather outdated chemical arsenal accumulated in the course of the 1940s to 1950s (the Soviet Union had completed the chemical rearmament of nerve agents more than 10 years later). There was a possibility of solving this problem in two ways: by a complete rearmament based on the most recent scientific-technical knowledge or by completing the program for the development of binary ammunition. General rearmament is, however, enormously economically and technically tedious, and in addition, it also includes the destruction of outdated stocks. On the other hand, even the introduction of binary technology does not facilitate this task. The United States spent considerable funds for the binary program, but the volume of the binary ammunition produced finally accounted for only 2% of the total chemical arsenal. Under these conditions, it was more advantageous to promote the general prohibition of chemical weapons and their total destruction under an international supervision [1]. In this way, the big world powers, under the leadership of the United States and the Soviet Union (and its successor, Russia), gave up their chemical arsenals and considerably restricted the proliferation of chemical weapons and the danger of their use, even in developing countries.

### 3.3. Operation-Tactical Reasons for Adopting CWC

Adopting the CWC was also supported by certain new views of the operational-tactical use. There were prevalent opinions that chemical weapons could be replaced to the full extent by the most modern conventional weapon systems (so-called intelligent weapons). Particularly restricting factors were held against chemical weapons—the dependence on climatic conditions (in spite of the fact that modern chemical formulae, ammunition and tools for attacking considerably reduced this dependence) and also the difficulty of the activity of military troops in a contaminated field. The efficacy of chemical weapons also suffered from the introduction of an advanced system of technical and organizational provisions for the anti-chemical protection of military troops and the population. However, from a solely military standpoint, chemical weapons are still the only actual available weapon system of mass destruction, which can be used for cases where a living force should be hit, but material objects should remain intact. On the other hand, there are a number of CWA that can cause the long-term contamination of the ground, equipment and technology or damage to sensitive materials. These negative impacts can result in requirements for subsequent decontamination.

### 3.4. Contemporary Risks of the Use of Chemical Weapons

The CWC was supposed to reduce the war threshold. What is the contemporary situation? The danger of the origination of a world war, *i.e.*, war between big powers, is ever persisting, if not immediate. General chemical disarmament has been implemented, except for a few cases (or at least all the facts suggest that this is the case). The historical experience shows that in the case of the danger of a world conflict or in the course of the conflict, the big powers are able to mobilize science and industry to produce a chemical arsenal at an even higher level. The immediate dangers are civil and ethnic wars, the frequency and intensity of which have demonstrated an increasing tendency over recent years. The accompanying phenomenon of these wars is the use of standard or improvised chemical weapons, the institutional control of which is enormously difficult. Any suspected ownership or use of chemical weapons in these wars can thus lead to enhancing the tension in international relationships (cases like Iraq or Syria).

A specific danger comes from international terrorism with the use of chemical weapons. The use of a poison as a tool for terror dates back even to ancient times and occurs in all the preceding historical eras, but in the time of scientific-technical development, it has appeared in certain new forms. A number of authors believe that the combination of terrorism and weapons of mass destruction, including chemical weapons, resulted in a modern form of terrorism, so-called ultra-terrorism [13,14]. Over the last half of the century, several hundreds of “chemical incidents” were recorded in the world, which were associated in a certain way with terrorism, but only a portion of them concerns the actual use of toxic substances [15]. Terrorists were interested in 60 different chemicals, mostly cyanides, organophosphates (including sarin and VX), harassing agents (tear gases) and natural poisons and toxins (such as, e.g., ricin). There are various methods of using toxic chemicals; they include direct contact with a chemical substance, scattering of an aerosol, poisoning of food, drinks and consumer products or the use of chemicals together with an explosive device [16]. The possibility of chemical ultra-terrorism is documented by the sarin attack carried out by the sect, Aum Shinrikyo, on the Tokyo subway in 1995 [17].

## 4. Contemporary Condition and Traditional Methods of Chemical Weapons Development

### 4.1. Nerve Agents As Most Important Chemical Weapons

Until recently, there has been a concept that the research and development of chemical weapons is a linear process, which necessarily leads to new, even more toxic CWA. Over the two years of World War I, the inhalation toxicity of CWA (mustard) was increased by a factor of 10 compared to chlorine. Over the next 20 to 40 years, the toxicity of new CWA (G and V nerve agents) increased by a factor of 100 compared to mustard. Since 1915, when chlorine was first used on a mass scale, the toxicity of CWA was thus increased by a factor of 1000 (Table 3). However, after V nerve agents, there had been no discovery of considerably more toxic substances and their mass introduction into armaments [18].

**Table 3:** Overview of the gradual increase in toxicity of CWA. LCt_50_, median lethal concentration; LD_50_, median lethal dose.

Introduction	CWA	LCt_50_ (mg·min/m^3^), Inhalation	LD_50_ (mg/70 kg), Percutaneous (Liquid)	LCt_50_ (mg·min/m^3^), Percutaneous (Vapor)
1915	Chlorine	10,000	-	-
1915 (1916)	Phosgene (diphosgene)	3,200	-	-
1916	Hydrogen cyanide	2,500	-	-
1917	H, HD	1,000	1,400	10,000
1918	L	1,000	1,400	5,000
1930s	HN	1,000	1,400	10,000
1930s–1940s	GA	70	1,500	15,000
1930s–1940s	GB	35	1,700	12,000
1940s	GD	35	350	3,000
1950s	VX	15	5	150

The currently existing nerve agents are exceptional synthetic warfare poisons. They are usually very stable and suitable for filling different types of chemical ammunition. They are well solubilized in organic solvents and penetrate into different natural, as well as artificial materials, thus causing unusual problems in the course of decontamination. The good solubility in water and resistance to hydrolysis makes their use for poisoning water sources or for other diverse purposes and sabotage (terroristic) possible. The spectrum of nerve agents given by the differences in the toxicity and physical-chemical characteristics makes it possible for the aggressor to select suitable substances under the conditions of different situations in the battlefield. Highly volatile substances, such as sarin, can be used in contact with the enemy to induce rapid inhalation poisonings without substantially limiting the activity of one’s own troops. On the other hand, V agents can be used (e.g., such as a filling in the warheads of operational-tactical rockets) for chemical attacks at different depths in the enemy’s position. Soman and other compounds of medium volatility, the vapors and aerosols of which can induce inhalation poisonings and the drops of which can efficiently strike the body surface, offer diverse methods of use. Symptoms of inhalation poisonings are manifested after several minutes, depending on the concentration (essentially immediately in the case of high doses), and in contrast to this, percutaneous poisonings (without warning manifestations) are characterized by a latent period, with the effect taking tens of minutes to several hours. A considerable characteristic of nerve agents is their cumulative action and relatively limited possibility for therapy with antidotes. In addition, the administration of antidotes can save the life of the persons exposed, but not their fighting capacity. The great psychological effect from the use of nerve agents is also of considerable importance [18].

### 4.2. The Problem of CWA with Increased Toxicity

For as long as several tens of years, military chemists have attempted to synthesize even more toxic nerve agents compared to those that have already been introduced into armaments. The synthesis of new substances is typically based on changes in particular groups of the basic chemical structure. The possibilities of G agents are thus limited, due to their relatively simple structure. In V agents, there are larger possibilities, particularly when changing the O-ester and thiocholine group, but even in this case, no compounds more toxic than VX or R-33 have been found. A further possibility is the synthesis of compounds based on mutual combinations of certain structural elements of G and V agents and possibly the introduction of certain new functional groups into the molecule. It is possible to believe that this approach was employed in the synthesis of the substances (A-230, A-232, A-234) of the Soviet binary program, NOVICHOK. The principle of these substances is, however, secret, and the admissible data are controversial. According to American sources, these are phosphorylated oximes from the group of *O*-chloro(fluorocarbiminoyl-*O*-[(2-chloro-1,2-dialkyl)ethyl]phosphorofluoridates [19]. Possibly more properly informed Russian sources report that the substance, A-230, is a liquid and stable methyl-{N-[1-(diethylamino)ethylidene]amido}phosphonofluoridate, the intravenous and percutaneous toxicity of which exceeds the toxicity of the VX by a factor of five to eight. Over several years, scientists synthesized more than a hundred structural variants of the substance, A-230, and tested them systematically. They also prepared its phosphate methoxy- and ethoxy-derivatives marked by the codes, A-232 and A-234. The toxicity of both substances is reportedly comparable with that of VX, but compared to the substance, A-230, they are less volatile and resistant to moisture. Ultra-toxic solid derivatives have also been reportedly synthesized, having a guanidine radical instead of the amidine one (substances A-242 and A-262) [20].

Relationships between atoms in molecules are strictly determined, and every sufficiently narrow interval of change in the molecular mass corresponds to a certain number of compounds. With increasing molecular mass, the structure and toxicity varies, but there is also a decrease in the probability that the substance will be volatile. The substances exerting considerably higher toxicity than nerve agents (VX or NOVICHOK) will thus be solid. The toxicity of solids is, however, also limited, and thus, the discovery of substances considerably more toxic than natural super-toxins (particularly botulinum toxin) is not expected. The fact should also be taken into consideration that even the introduction of CWA with higher toxicity does not result in the proportional enhancement of other combat characteristics. For example, the increase in the toxicity of a CWA by a factor of 10 corresponds to about a doubled area for the effective contamination with contemporary types of chemical ammunition [18].

### 4.3. Problem of Overcoming the Anti-Chemical Defense

The increase in the toxicity of CWA is not the only possibility for how to enhance the efficacy of chemical weapons. Every new CWA has been expected to be able to overcome the anti-chemical defense of the potential enemy and to force him to solve new and complex problems regarding detection, the protection of respiratory organs, as well as the body surface, decontamination and therapy. These requirements were obviously also followed in the development of the NOVICHOK program. In the case of their possible use, an insufficient sensitivity and selectivity of the existing means of detection is to be expected, except for those based on biochemical reactions. In the case of the phosphorylated oximes already discussed, the contemporary therapy for poisonings with nerve agents based on AChE reactivation could possibly fail.

#### 4.3.1. Penetration of CWA through Isolation Barriers

The attempts to improve second generation chemical weapons resulted in searching for liquid CWA, which could easily penetrate through the anti-chemical isolation polymer barrier and also eliminate living forces through percutaneous action. The research was focused on organophosphate nerve agents, which are more persistent than sarin and more volatile than VX. The military laboratories of the United States synthesized compounds marked IVA (intermediate volatility agents), the structure of which is most likely to contain certain typical elements of the G agents (fluorine), as well as the V agents (2-dialkylaminoalkyl group). In the special literature, they are thus marked by the code, GV (or GP). Their disadvantage is their low stability, but this problem was solved by the design of the binary ammunition (IVA-2). The Soviet Union and Iraq introduced soman and cyclosarin, respectively, with similar effects.

#### 4.3.2. “Mask Crushers”

As early as World War I, the German army elaborated the concept of “mask crushers” based on the use of solid organic compounds of arsenic (diphenylchloroarsane, diphenylcyanoarsane, later adamsite) exerting irritating effects on the airways. Even negligible concentrations of these substances in an aerosol easily penetrated through protecting charcoal filters and forced soldiers to remove protective masks, thus facilitating the effects of the concomitantly used lethal species of the CWA. Because of this, new filters with a special anti-smoke insert have been introduced. Further military research was focused on overcoming the protective filters by using volatile substances with lethal effects. Already in the 1930s, choking agents were studied that exerted an enhanced capability for penetration, such as, for example, trifluoronitromethane (the fluorine analogue of chloropicrin), trifluoronitrosomethane, hexafluoroazomethane, chlorotrifluoride and perfluoroisobutene. These substances are highly toxic (for example perfluoroisobutene is more toxic than phosgene by a factor of eight), but their disadvantage is in the slower onset of their effects. The “mask crushers” can also be considered to include simple toxic gases, such as carbon monoxide, arsane, phosphane or cyanogen chloride. Still, the best solution for protection from the effects of the inhalation of “mask crushers” is the use of isolation devices.

#### 4.3.3. Replacement of Nerve Agents

In the past, there were tactical, as well as technological reasons for compounds that could completely replace the existing organophosphorus nerve agents. Attention has long been given to carbamates, which exert a similar pattern of intoxication, but in the course of therapy, it is (in contrast to organophosphates) impossible to employ antidotes based on AChE reactivators. However, volatile carbamates exert a low toxicity. The most toxic carbamates are quaternary arylcarbamates (for example, T-1123), which are solid and, thus, cannot replace nerve agents in standard chemical ammunition. They are, however, ideal as poisons for the purposes of diversion or sabotage.

An integral part of military programs has also been the study of substances with unusual mechanisms of action compared to traditional CWA. From this standpoint, certain highly toxic, industrially available and easily acquired organophosphorus bicyclic compounds, particularly bicyclophosphates, have been of interest. The mechanism of their toxic effect is not completely understood, but in accordance with the most recent findings, in contrast to G and V nerve agents, it is based on the inhibition of the neurotransmitter, GABA (γ-aminobutyric acid). There is the assumption that toxic bicycloorthocarboxylates and norbornanes also interact with GABA-receptors. The advantage of this group of substances for military purposes is, in particular, their difficult detection and the unavailability of a specific therapy.

#### 4.3.4. Tactical Mixtures

The physical and chemical characteristics of CWA-filled ammunition (such as, e.g., the stability) have formerly been typically adjusted with suitable admixtures. Certain CWA (mustard, lewisite, soman, R-33) were applied in a thickened form, which improved the ballistic characteristics of the ammunition and made possible the effective transfer of the agent into combat conditions. In addition, auxiliary components were searched for to accelerate the absorption of CWA into the organism. It is, for example, known that dimethyl sulfoxide increases the ability of CWA to penetrate through the skin several times over. Similar penetration characteristics are also known for substances that are currently subjected to intensive medicinal, pharmaceutical and cosmetic research (for example, 1-dodecylazocycloheptane-2-one) [21].

In the past, mixtures of CWA were also proposed that exerted unusual toxic effects and were associated with the enhanced tediousness of chemical analysis and the use of protective tools, decontamination and therapy. These were, for example, mixtures of mustard with O-mustard, mustard with lewisite, mustard (or lewisite) with diphosgene, mustard with chloroacetophenone, hydrogen cyanide with cyanogen chloride or BZ agent with liquid, irritating 1-methoxy-1,3,5-heptatriene. There were also attempts to combine nerve agents with the aim of enhancing their volatility or capability of penetration through the skin. An example is a mixture of sarin with cyclosarin. Some combinations of organic phosphorus compounds may also have a synergistic effect [22]. A special chapter of the history of chemical weapons comprises mixtures of CWA with natural toxins or with infectious agents (for example, mustard with aflatoxins or mustard with anthrax).

### 4.4. Natural Poisons and Toxins

Natural poisons and toxins are a very extensive group with respect to the number and structural types of substances, presenting a basis for toxic (chemical, biochemical, toxin, biological) weapons. Plant poisons, animal poisons, bacterial toxins, toxins of cyanobacteria and algae or fungal poisons (mycotoxins) have been employed by man for fighting and hunting purposes since the earliest times. Some of them, such as ricin (W), botulinum toxin (X) or saxitoxin (TZ), were formerly proposed as standard fillings of military ammunition, and some others (palytoxin, batrachotoxin, tetrodotoxin) were subjected to intensive military research. There are several differences between toxins and traditional chemical weapons. Toxins have higher molecular weights (as solids); most of them are odorless and not dermally active, and a number of them produce immune responses. Their attractiveness results from their extreme toxicity and the possibility of their industrial manufacturing with the use of the most modern technologies (although difficult on a large scale). From the operational-tactical standpoint, natural poisons and toxins are essentially easy to use, some of them as inhalation poisons. For example, the lethal inhalation concentration (LCt_50_) of a botulinum toxin aerosol is of 0.02–0.1 mg·min/m^3^, which exceeds the toxicity of sarin vapors by a factor of about 1,000 on average. In the case of these substances, detection, protection and therapy are complicated, due to the diverse forms of toxic effects, and in addition, their military use is difficult to demonstrate, due to their possible natural abundance in the target area. By way of example, it is possible to mention a unconfirmed suspicion that at the end of the 1980s, the Soviet army used a chemical weapon in South-East Asia based on mycotoxins from the group of trichothecenes (toxin T-2), in the form of so-called yellow rain [23].

### 4.5. Binary Chemical Ammunition

Binary ammunition is a special chemical reactor containing two relatively non-toxic components, which react with each other in the course of the flight to the target, producing CWA. The binary system offers a number of advantages. It particularly increases the safety in the manufacturing, storing, transfer, use and destruction of chemical weapons. The shelf-life of the chemical ammunition is considerably prolonged. Binary technology makes possible a simple exchange of precursors in accordance with operational plans and the tactical situation. In binary ammunition, it is also possible to use CWA, which are less suitable (instable) for filling unitary ammunition, or possibly in different tactical mixtures. The principle of binary chemical weapons, however, has drawbacks, such as, e.g., a reduction in the useful mass of the filling or a decrease in the transfer coefficient of the CWA to the action conditions. The chemical reaction in the ammunition is then accompanied by heating the reaction mixture, which can result in a decomposition of the CWA produced, particularly in areas with a hot climate. In shooting directed at close targets, the reaction time, and thus, also, the reaction yield, is reduced. The synthesis of solid CWA is extremely difficult in binary ammunition. Tests of binary chemical ammunition demonstrated its efficacy to be at least 1.5–2 times lower compared to single-component ammunition. This means that in the course of its design, the CWA used should be more toxic by a factor of at least 2–3 than V agents [18]. There is a possibility that this condition was adhered to within the framework of the NOVICHOK program. In addition to the improvement of the binary system, there is the possibility of the development of multi-component ammunition (comprising three or more components), which calls for even higher requirements for the characteristics of CWA and the implementation of the CWC. They were developed as alternatives and less advanced concepts for binary weapons. For example, Iraq developed “binary” aerial bombs, warheads for tactical missiles and artillery shells filled with only one component (a mixture of isopropyl alcohol with cyclohexanol). The second component (methylphosphonyl difluoride) was added just before combat use. After mixing the two components, mixtures of sarin with cyclosarin were obtained.

### 4.6. Non-Lethal Chemical Weapons

#### 4.6.1. Characteristics of Non-lethal Chemical Weapons

The pressure for the development of the conduction of “non-bloody and humane war” has recently been ever increasing as a part of a wider discussion about the revolution in the matter of military science and technology. Non-lethal weapons are currently a common supplement to conventional weapon systems [24]. These are weapons explicitly designed and developed for making persons unable to fight or for their elimination from action with a lower probability of fatal (or permanent) injury or for the elimination of armaments. A part of these technologies are also chemical weapons, the action of which is based on non-lethal (or lower than lethal) toxic effects.

Under currently existing conditions for the means of anti-chemical protection, a situation can be encountered for which the destruction of living forces by lethal species of the CWA would be non-realistic. There will be a higher importance of temporarily incapacitating effects (immobilization, binding) with the help of non-lethal CWA, which act even at extremely low concentrations. This group includes very different physiologically active substances with a different nature to the toxic effects, in which the effective (incapacitating) doses are lower by a factor of at least 100 compared to lethal doses. This is different, for example, from the use of nerve agents. Of course, it should be emphasized that from the toxicological standpoint, the classification of chemical substances as lethal and non-lethal is problematic (see the definition of poisons by Paracelsus). The safety of a chemical substance can only be established based on experiments in animals and only estimates can be obtained for humans. The estimate does not take into consideration individual sensitivity to chemical compounds and particular conditions of their use.

The genetic association between non-lethal chemical weapons and incapacitating agents, which has always been a part of military chemical arsenals, cannot be omitted. These were first harassing substances—tear gases and respiratory irritants; the psychoactive BZ agent or, possibly, other anticholinergics based on glycolates occurred later in chemical arsenals. The military specialists were, however, also interested in other groups of substances with effects causing mental and physical disability, such as, e.g., sympathomimetic (LSD-25), dissociative anesthetics (phencyclidine, ketamine), narcotic analgesics (fentanyl and its derivatives), neuroleptics, tremorgenes (tremorine) or emetics (apomorphin, staphylococcal enterotoxin B). The ideal incapacitating substance is highly efficient (in a dose of the order of magnitude of μg/kg and below) and exerts rapid and short-term action (several minutes). Its effect is reversible and also expectable, depending on the dose. There is also an obvious requirement for its good stability upon storing and handling [25]. These requirements considerably restrict the group of potential candidates, but also indicate the direction for the future research and development of incapacitating agents.

So-called calmatives are of special interest from the military standpoint (knock-out gases, immobilizing agents), which hit the organism at different levels of the central nervous system, compared to irritating substances, and induce changes in the mental condition and behavior. Many of these compounds are quite commonly used in human and veterinary medicine, such as, e.g., sedatives, hypnotics, anesthetics, analgesics, myorelaxants, anxiolytics, antipsychotics, antidepressants, *etc.* However, specialists are interested in benzodiazepines affecting GABA-receptors, alpha_2_-antagonists with bonds to alpha_2_-adrenergic receptors or opioids, with enormous effects on opiate receptors. The enhanced interest in synthetic opioids induced an antiterrorist intervention of Russian safety forces in October, 2002. To rescue the hostages in the Moscow theatre, Dubrovka, they probably used an aerosol containing a mixture of carfentanil and remifentanil [26]. Synthetic opioids are considered as extremely potent analgesics and anesthetics with a very rapid onset of action. For example, the narcotic effect of carfentanil is higher by a factor of 10,000 compared to morphine and by a factor of 100 compared to fenantyl. It is used in veterinary medicine to immobilize big mammals (elephants, bears) in the form of special gun projectiles and aerosol generators. However, the effect can be unexpected and can endanger life. This was also supported by the result of the antiterrorist intervention in Moscow, where about 130 (*i.e.*, 15%) of the persons present died from poisoning.

It should be emphasized that the category of non-lethal chemical weapons finds no support in the CWC. CWC defines only chemical weapons, which means toxic chemicals and their precursors. Toxic chemical means any chemical that can cause death, temporary incapacitation or permanent harm to humans or animals through its chemical action on life processes. This means that any toxic chemical intended for use in war (including internal armed conflicts) is a chemical weapon.

#### 4.6.2. Riot Control Agents

Certain characteristic features of non-lethal chemical weapons are also encountered in so-called riot control agents (RCA). This can cause confusion, because RCA have a separate definition and regimen under the CWC. RCA means any chemical not listed in a schedule that can rapidly produce sensory irritation or disabling effects in humans, which disappear within a short time after the exposure. RCA may be used for purposes relating to law enforcement including a domestic riot control. Each State Party of CWC undertakes not to use RCA as a method of warfare.

**Figure 1:**
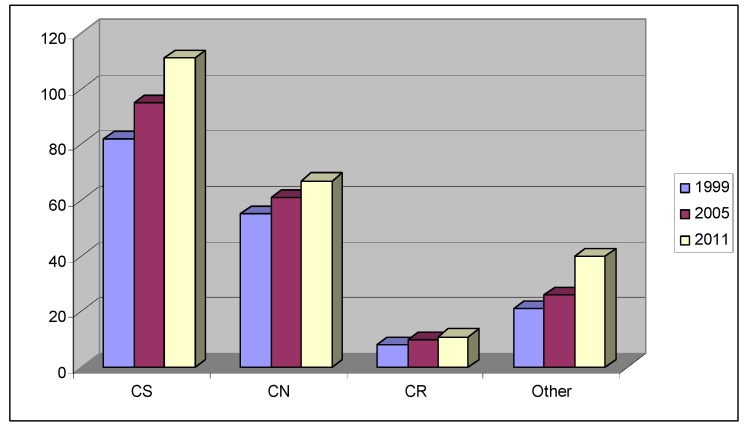
The number of countries of the Chemical Weapons Convention (CWC) declaring different types of riot control agents (RCA) in 1999, 2005 and 2011.

The traditional representatives of these means are harassing agents (tear gases). As shown in Figure 1, CS and CN agents enjoy the highest interest throughout the world, but other agents are also finding their application stepwise (CR agent, capsaicin or its synthetic homologues, such as, e.g., pelargonic acid vanillylamide). The development of this group of physiologically active substances has not yet been completed. It is known that the substances with extremely irritating effects include many esters of plants of the genus, *Euphorbia*. Some of them (for example, resiniferatoxin) have already been completely synthesized in laboratories [27]. A tendency to accept RCA, as well as other substances with mentally and physically incapacitating effects (including the fentanyl derivatives used by Russian special corps in the Moscow incident), has recently occurred. This may be against the purpose of the CWC.

#### 4.6.3. Malodorants

A special case of non-lethal chemical weapons and potential RCA are agents that cause intense odor (so-called malodorants). This is not a new technology. So-called stink bombs have been known since the Middle Age, and in World War I, they served to mask the presence of CWA. There is information from the mid-1960s concerning the development of malodorants as effective components of chemical weapons and means for suppressing riots in areas inhabited by certain ethnic groups. The contemporary programs are oriented toward the research of malodorants with enormous effects on human chemical sensors and toward defining the nature and measure of the response. A number of known and available chemicals, which are present in the defensive secretion of certain animals, can be used for police or military purposes. For example, the secretions of skunks (*Mephitis mephitis* and related species) contain unbearably smelly thiols (2-butene-1-thiol, 3-methyl-1-butanethiol), their thioacetates and alkaloids, which are easy to synthesize, chemically modify and industrially manufacture. Chemical compounds having structures close to cadaverous poisons (putrescine, cadaverine) or human pheromones that release body odors are other examples.

The use of malodorants not based on toxic effects (in certain substances, however, toxicity was described) can meet the criteria for the definition of RCA by CWC (they are able to rapidly induce irritation of sensory organs). Consequently, they cannot be used in a war. The development and introduction of natural malodorants is also problematic in terms of the BTWC. In spite of this, malodorants can become the basis of an attractive modern concept for the conduct of war. They also can mask the development and production of weapons and ammunition (delivery systems) in which other chemical agents can also be used, including lethal ones [28].

### 4.7. Toxic Industrial Chemicals

The term toxic industrial chemicals (TIC) refers to a variety of chemicals used or produced by industry that can have significant impacts on human health if released into the air or water. According to the CWC, any TIC can be considered a chemical weapon, if it is designed or used for military purposes. In the past, much of the CWA consisted of TIC (chlorine, phosgene, hydrogen cyanide, chloropicrin, *etc.*) or were developed in the context of civilian research and development (for example, nerve agents). The main risk of TIC’s is that they are manufactured and stored in vast quantities and can be relatively easily used in war or domestic armed conflicts. The general limitation of the production and consumption of TIC is extremely difficult for various reasons.

The group of TIC’s also includes some unusual compounds that may be of interest to experts in chemical weapons. These are, for example, dioxin (2,3,7,8-tetrachlorodibenzo-p-dioxin, TCDD) and its analogues. TCDD is well known as an impurity in the herbicides (for example, Agent Orange) used in the war in Indochina. It is also known in connection with an industrial accident in 1976 in Italy (Seveso). This is an extremely toxic synthetic agent capable of alkylating various cellular polymers, including DNA. Some clinical symptoms of poisoning are similar to mustard gas. TCDD has a long latency period, and poisoning is difficult to treat. It is ideal for ecological war, because it can cause long-term and dangerous contamination of large areas, even when used at relatively low concentrations.

## 5. New Technologies in the Development of Chemical Weapons

A rule has usually been valid in history that any weapon vanished only if it was replaced by a new and even more effective one. This probably mostly holds for chemical weapons. In addition, the possible exhaustion of the possibilities for technology of weapons of mass destruction does not mean a decrease in demand. The control of chemical weapons and the possibilities of their further development are associated with the term “dual use technology” [29]. This is technology involving civil and, simultaneously, military applications. These are materials, programs and knowledge used in peaceful applications, which can, however, also be used in the production of weapons. The dual use technologies can considerably facilitate and speed the manufacturing of standard chemical weapons. They make possible a better understanding of biological systems, and the results of the study can be then used for the development of new, still unknown types of chemical weapons. With the help of these technologies, it is also possible to propose new, more effective forms of toxic substance applications. Their effects on adherence to international legal standards, including the CWC and BTWC, are thus negative [30].

In association with the development of chemical weapons, the term biochemical weapons has recently been used, which reflects the fragile boundary between chemical and biological weapons (Table 4) [31]. It is tightly related to the term biochemicals, which are biologically active chemical compounds or compounds produced by a specific chemical mechanism in a living organism. If traditional chemical weapons are the products of chemistry and the chemical industry, then biochemical weapons should analogously belong to the domain of biochemistry and the pharmaceutical industry [32].

**Table 4:** The relationship between chemical and biological weapons.

Category	CWC	BTWC	Poisons	Infectious Agents	Biochemical Weapons
CWA	+		+		
Chemical drugs	+		+		+
Bioregulators	+		+		+
Toxins	+	+	+		+
Biological weapons		+		+	

### 5.1. Technology for Acquiring New Biologically Active Substances

#### 5.1.1. Combinatorial Chemistry

The traditional research and development of new physiologically active substances is enormously expensive, tedious and offers unsure result. In laboratories, large quantities of different compounds are produced; their efficacy is tested, and the chemical structure is searched for, which could be suitable for the further development of a medicine or other product. This will be changed in a revolutionary manner in the future. The synthesis of peptides based on changing amino acids and the development of “parallel synthesis” techniques or “split-and-pool” synthesis were established for so-called combinatorial chemistry, which makes it possible to systematically combine different chemical construction blocks and, thus, to obtain big files (libraries) of structurally close compounds. These libraries can then be used with the technique termed “high-throughput screening” to identify specific compounds, depending on the effect required. There is a consideration that the probability of finding a new biologically active compound is increased by a factor of an order of magnitude of about 1000. These possibilities were also extended by the discovery of a new method named “click chemistry”. The risks of using combinatorial chemistry and associated procedures for the development of toxic compounds with military potential are difficult to estimate. The technology is particularly suitable for the synthesis of compounds with a molecular mass of about 700 Dalton, which is more than the value in traditional CWA. It can be used for the development of incapacitating agents of a new generation [30].

#### 5.1.2. Protein Engineering

Protein engineering, first described in early 1980s, deals with the targeted production of proteins, particularly enzymes. It also has penetrated into the pharmaceutical industry, including the field of the development of protein hybrid (fusion) toxins suitable for therapeutic purposes. Protein toxins occurring in nature as animal and plant poisons are capable of a specific action on cellular metabolism, and thus, they are dangerous, even at extremely low doses. Based on protein engineering, it is possible to combine the binding and catalytic domains of two different protein toxins and, thus, to produce a hybrid toxin. On laboratory, as well as industrial scales, it is possible to obtain hybrid or modified protein toxins with enhanced lethality, with a greater extent of effects and resistance to detection, diagnosis and therapy. For example, the extremely toxic, but under common conditions, less resistant, botulinum toxin presents a highly lethal hybrid toxin in combination with stable and resistant staphylococcal enterotoxin, which is also resistant to heat and to adverse media. By a combination of the catalytic domain of tetanus or shiga toxin with the binding domain of anthrax toxin, a hybrid toxin is obtained, affecting a wider spectrum of monkey cells compared to the original toxins [33,34,35].

### 5.2. Technology of Manipulation with Biological Systems

#### 5.2.1. Research of Psychoactive Substances

In the early 1990s, scientists acquired new knowledge on protein neurotransmitter receptors bound to the outer neuron membrane with the help of the techniques of molecular biology and genetics. In contrast to simple acetylcholine, some of these neurotransmitters have a complex peptide structure with short amino acid chains. For example, in a study of the sleep disorder called narcolepsy, in the 1990s, new neurotransmitters, hypocretins (orexins), were discovered, which are produced by hypothalamus cells [36]. In the mammalian hypothalamus, a neuropeptide hormone, oxytocin, is also produced, which is associated with social behavior and reproduction (the so-called hormone of love and trust). One of the possible forms of its pharmacological application is aerosol [37]. Knowledge from the area of neuropeptides can also be obviously used for the development of new agents as potential non-lethal weapons [38]. Knowledge is also being extended concerning the effects of psychoactive substances, which were formerly considered as standard or potential components of military armament. It was, for example, found that the BZ agent and its analogues are bound to five sub-types of muscarine acetylcholine receptors, which have different functions in the brain. This explains their pleiotropic and variable effects [39].

#### 5.2.2. Synthesis of Bioregulators

Bioregulators are natural organic compounds that regulate different cellular processes in multiple organic systems [40]. They regulate the division and differentiation of cells, blood pressure, heart activity, respiration, muscle contractions, body temperature or sleep and affect mood, emotion and immunity functions. Bioregulators are effective, even at extremely low concentrations, and act essentially immediately. At higher than normal doses, they can induce different toxic effects or even death. The enhanced interest of biotechnological and pharmaceutical institutions and companies in bioregulators is associated with the progress in the field of molecular biology and biotechnology and with the development of a new generation of pharmaceuticals with increased specificity and efficacy. Based on estimates, about 10,000 bioregulators exerting different chemical structures and functions are active in the body of homoeothermic animals. In addition, many of them contain not so large structural fragments exerting the same activity of the original bioregulator. These fragments, which occur independently in nature, can be used as initial materials for the synthesis of analogues. The number of these compounds, where some of them have toxicity comparable with nerve agents, is ever growing [39].

The idea for the development of chemical (biochemical) weapons based on bioregulators occurred as early as the pioneering era of the United States’ center for the development of biological weapons in Fort Detrick, but its implementation was impossible that time, due to the lack of suitable technology. In spite of this, the Ministry of Defense financed several research and development programs as early as the 1980s, focused on biotechnology, including a project for the development of biochemical (hormonal) weapons, including bioregulators [41]. That time, the Soviet Union reportedly included bioregulators in an offensive program for the development of biological weapons [42]. The bioregulators are currently considered to be prospective incapacitating (non-lethal) weapons, and their research is being performed by scientific teams throughout the world (as, for example, in the United States, Great Britain, France, Russia, China, Israel). The most recent progress in the field of molecular biology and biotechnology essentially simplified the preparation of bioregulators, which would be formerly available only in negligible amounts from natural sources. The methods of gene engineering make possible the production of bioregulators, even in amounts sufficient for filling ammunition. Bioregulators can thus present a new dimension in the conduct of modern war. Their accepting by certain countries as non-lethal weapons of a new type can also legitimize the use of toxic substances in war [43]. They can also be considered as potential terrorist weapons [44].

Endorphins and enkephalins can also be of importance for military purposes. These are opioid polypeptides excreted from the hypophysis in the course of physical stress, which can block pain or induce feelings of peace of mind (so-called “hormones of happiness”). One can speak about neurokinins, for example, substance P, which is a peptide participating in the transfer of nociceptive impulses and controls adaptation stress mechanisms. The regulation peptide, cholecystokinin (CCK), can also be of interest, which induces strong emotional reactions to panic. There are several natural forms of this peptide with different numbers of amino acids (such as, for example, CCK-8, CCK-33), but with a high physiological activity, and also having shorter fragment molecules of this peptide, so-called CCK-4. Further research resulted in the synthesis of CCK-4 analogues, which are characterized by their enhanced affinity to receptors, metabolic stability and ability to overcome the hematoencephalic barrier. An example of high-activity peptide bioregulators is also endothelin, which increases arterial pressure and reduces heart beat frequency. It is close to sarafotoxins with respect to its structure and function, which were isolated from snake venom (*Atractaspis engaddensis*). Certain metabolites of arachidonic acids (prostaglandins, prostacyclins, thromboxanes, leukotrienes) or the bioregulator of the phospholipid structure, known as the thrombocyte activating factor, are also effective regulators of the cardiovascular and respiratory systems or mediators of different pathological conditions (inflammations, allergy, anaphylaxis, shock). The disadvantage of bioregulators is their low stability. Endogenous bioregulators are rapidly degraded in the organism in the presence of catalytic ferments. Due to this, the use of bioregulators in the form of formulae containing efficient inhibitors of these ferments is considered. However, these regulators alone can be highly active substances causing pathological effects [18].

#### 5.2.3. RNA Interference

RNA interference is a process regulating transcription and intracellular gene expression in such a way that certain fragments of the double-helix RNA interfere with the genes expressed. The first experiments were performed by American scientists as early as in 1990. They genetically modified petunia by introducing the gene for enhancing the level of the enzyme participating in the synthesis of the violet dye. This technology was later tested in other organisms and introduced into pharmacy and medicine as a promising method of treatment of still non-curable diseases at the molecular level. However, RNA interference can also be abused, for example for the preparation of genetically-modified toxins or for affecting an organism’s immunity. There is, for example, a possibility of abusing RNA interference for the targeted affecting of genes present in specific ethnic groups only [45]. This can serve as a basis of modern chemical (ethnic) weapons [46].

### 5.3. Technology of Manufacture and Use of Toxic Substances

#### 5.3.1. Chemical Microprocessor Devices

In the 1980s, chemical microtechnology was developed, which induced revolutionary changes in chemical and pharmaceutical industries. Compared to standard chemical operations, the miniaturized devices are safer, more rapid, more selective and more effective with respect to the energy requirements. These are automatized devices with micromixers, miniaturized heat exchangers and reactors of a volume of about 2 L. The capacity of a line, the installation of which a room of 30 m^3^ is sufficient, provides several kilograms of the pure product per hour. Chemical microprocessors for the manufacture of pure chemical and pharmaceutical products are currently quite commonly used in big global companies [47]. There is a risk that this technology will also be employed for the manufacturing of toxic compounds important for military purposes, which will attenuate the control mechanisms of the CWC. According to certain specialists, it is less suitable for the manufacturing of classic CWA [48], but it may be very progressive for the manufacturing of toxic agents of a new generation (including prospective non-lethal chemical weapons).

#### 5.3.2. Microencapsulation

Special techniques have also been developed that enhance the CWA stability and make possible the penetration of the agents and their controlled release into an organism. One of these techniques is microencapsulation based on covering the CWA in a micro-droplet with a biologically degradable polymer envelope. This is the same technique as that used in the manufacturing of pharmaceutical products. The microcapsules can be employed in different ways. For example, during the explosion of the ammunition, they produce an aerosol cloud and penetrate via the respiratory tract into the organism, where their envelope is dissolved, releasing the CWA. This technology can also be easily used for the development of new forms for the application of non-lethal chemical weapons, and thus, it is being intensively studied [49]. Unstable biologically active agents, which would otherwise be quite unsuitable as chemical weapons, can also be employed based on the microencapsulation technique.

#### 5.3.3. Nanotechnology

The effects of nanoparticles on living organisms result from certain interesting characteristics. Nanoparticles exert high chemical and catalytic activities, which have not been observed in larger particles of the same substance. In the medium (in air), nanoparticles form high concentrations, even at a relatively low amount of the substance dispersed. Nanoparticles can easily penetrate into the human organism through all of the means of entrance (for example, by inhalation, percutaneously, by ingestion, through the eye barrier) and invade any organs and tissues, including the central nervous system. Nanoparticles can exert serious (even lethal) effects on humans, which is manifested by the clinical pattern of a disease that is unknown to physician or masked by other clinical symptoms. A special case is given by nanoparticles smaller than 50 nm, which can cause genotoxic effects due to their capability of penetrating into the cellular nucleus [50].

The use of nanoparticles for military purposes is not quite new. For example, solid organic compounds of arsenic (“mask crushers”), used in World War I through the explosion of ammunition, produced (by so-called self-condensation) irritating aerosol, which contained aggregates with a particle size under 100 nm. Toxic smokes were later produced with the help of thermogenerators and smoke pots. In the Vietnam War, the American army used the harassing CS agent [51] in the form of the formulae, CS1 and CS2. The formula CS1 is a finely ground powder of the effective compounds with an admixture of 5% pyrogenic silica gel (Cab-O-Sil, particle size of 5–50 nm). It is suitable in all the types of weapons with a highly explosive charge or in special dispersing equipment. CS2 is essentially the formula CS1 microencapsulated with the help of hexamethyldisilazane, which is resistant to hydrolysis and high temperatures. Both formulae are currently common parts of riot control ammunition. Scientists have also been recently interested in the preparation of nano-materials based on biologically active peptides [52,53] and other substances. These technologies are prospects, for example, medicine, but can also be used for the production of new forms of bioregulators, calmatives and other groups of toxic substances for military purposes.

## 6. Conclusions

The existence of chemical weapons is shown not to be generally associated with a particular historical era, though their development is, of course, conditioned by the level of science and technology. In a certain sense, chemical weapons have a “universal nature” in history. The acceptance and implementation of the CWC is an important human action, the topical nature of which has not been realized, even the factual destruction of all of the stocks of chemical weapons. The CWC should be open to the future and provide protection from scientific and technological development. In accordance with many parameters, chemical weapons based on traditional technologies have achieved the limit of their development. There is, however, a big potential for their further development based on the most recent knowledge of modern scientific and technical disciplines, particularly at the boundary of chemistry and biology. The risk is even higher due to the fact that already today, there is a general acceptance of the development of non-lethal chemical weapons (or RCA) at a technologically higher level. It is thus possible to assume that in the future, new forms of the accumulation of stocks of chemical (biochemical) weapons will occur, which will be produced by the virtual synthesis of new toxic substances from unmonitored chemical agents. Their characteristics will be studied on the microscale, and computerized models will be used to simulate the possibilities of their military use. In the future, the chemical arsenal will be based on the accumulation of important information from the fields of chemical, biological and toxin weapons. Data banks obtained in this way will be hardly accessible and the risk of their materialization will persist. In practice, this means that in the future, the control mechanisms should include declarations of relevant scientific and research programs and their control [54]. This is, however, very problematic, since free scientific investigations should not be prevented by any control mechanism.
